# Intra-specific structural variation among Hawaiian *Hoplothrips* (Thysanoptera, Phlaeothripidae), with ten new synonymies and one new species

**DOI:** 10.3897/zookeys.722.22131

**Published:** 2017-12-14

**Authors:** Laurence A. Mound

**Affiliations:** 1 Australian National Insect Collection, CSIRO, Canberra, ACT 2601, Australia

**Keywords:** Synonyms, fungus-feeding, sexual dimorphism, polyphenism

## Abstract

Most of the 16 fungus-feeding species described from the Hawaiian Islands and now placed in the genus *Hoplothrips* were based on very few and incomplete specimens. The descriptions were published long before any studies on the biology and structural variation of fungus-feeding Phlaeothripinae. Ten of these species are here placed into synonymy, and doubts are expressed concerning the identity of some others. One new polymorphic species is described and compared to a species known only from Florida. In the absence of comprehensive studies on the *Hoplothrips* fauna of North America, there is little evidence of endemicity or radiation on Hawaii within this genus.

## Introduction

The genus *Hoplothrips* in the insect Order Thysanoptera comprises 130 described species (ThripsWiki 2017), almost all of which live on dead branches of woody angiosperm trees. The larvae and adults of these thrips feed either on fungal hyphae, or possibly the breakdown products of fungal decay (Kobro and Rafoss 2006). They often form considerable colonies, involving all life stages. Presumably in response to the transience of this fungal habitat, many species produce winged, dispersive, adults as well as wingless (or wing-reduced) adults. This wing dimorphism involves, in both sexes, considerable differences in body form and chaetotaxy. Moreover, there is great variation in adult body size, both within and between colonies, and presumably this too is related in some way to variation in the quality and quantity of fungal growth. Many *Hoplothrips* species also exhibit considerable sexual dimorphism, with size-associated allometry resulting in large males having enlarged fore femora and fore tarsi bearing an enlarged tooth. As a result, large males look very different from small males, and small males resemble females in body form. Crespi (1986, 1988) has demonstrated that this sex-related structural variation is associated with behaviour, including male/male competition. These patterns of structural variation within individual species, involving wing and sexual dimorphism, together with body size polyphenism, often cause problems for species recognition and generic diagnoses (Mound 2005).

In Hawaii, most descriptive work on *Hoplothrips* dates from 1910, with a few more species added in 1928 and 1936; these descriptions thus predate any serious appreciation of the intraspecific structural variation that is now known to be common in members of this genus. Moreover, that early work predated any appreciation of the extent to which the Hawaiian insect fauna includes adventive species (Beardsley 1962). The objective of the present study has been to reassess the taxonomic significance of the 16 species of *Hoplothrips* described from the Hawaiian Islands, based on a re-examination of the original specimens, together with more recently collected material acquired particularly during June 2016 (Mound et al. 2017).

## Bagnall thrips collection

Of the 16 species of *Hoplothrips* listed from the Hawaiian Islands, 11 were described by Bagnall (1910) based on specimens collected by RCL Perkins between 1890 and 1900. Bagnall stated that this material “consists chiefly of about seven dozen dried and mounted specimens, though later a small collection in alcohol was submitted.” For subsequent workers, it is essential to remember that most of the descriptions by Bagnall were based on these dry and shrivelled, card mounted, specimens, and such descriptions must thus be interpreted with caution. Moreover, as Bagnall himself pointed out, six of his 11 species were based on single individuals, four of which had no antennae (the antennae of a fifth holotype also no longer exist). In 1967, the few remaining carded specimens from the original series were examined at the Natural History Museum, London, and many were found to be covered in a long-established layer of white fungus. These specimens were all cleaned of fungus and then slide-mounted into Canada balsam (Mound 1968). From this historical background, it can be understood why useful information concerning these 11 species is at best meagre, whether from the original publication or from the few remaining specimens. Certainty about species identity cannot be secure when based on such specimens that lack legs, antennae and most setae. Moulton (1928, 1936) had available to him from Hawaii more extensive series of specimens, but in comparing his new species he sometimes gave little consideration to morph differences, and his comparisons to the Bagnall species were based on the words and drawings in the 1910 publication, not on personal observation of any Bagnall specimens.

## Hawaiian *Hoplothrips* radiation

The studies presented here have led to some major conclusions. Of the 16 described species, 10 are here newly synonymised. As a result, it is concluded that there is little evidence of any major radiation within the genus *Hoplothrips* on the Hawaiian Islands. Moreover, the suggestion by Bagnall that different islands support different *Hoplothrips* species is not supported by the available evidence. Earlier studies were based on the *assumption* of endemicity amongst the Hawaiian thrips fauna. However, the species discussed below under the name *dubius* cannot be distinguished satisfactorily from a species recorded commonly in North America; some of the other species also seem likely to represent recent introductions. In this genus, species taxonomy worldwide is difficult and confused. Despite 130 species being listed in the genus (ThripsWiki 2017), identification keys are available only to 11 species from Illinois (Stannard 1968), 8 species from Britain (Mound et al. 1976), 12 species from Germany (Schliephake and Klimt 1979), and 17 species from Japan (Okajima 2006). In contrast, Mound and Marullo (1996) listed 34 *Hoplothrips* species from the Neotropics, and Stannard (1957) listed 32 species that are now placed in this genus from North America, although the *Hoplothrips* fauna of western USA is clearly poorly known (Hoddle et al. 2012). Without comparative studies on the species from the American mainland there is no possibility of evaluating the status of the species recorded from Hawaii. One remarkably polymorphic new species of this genus is described below, and this shares particular character states with species from the Americas. The Hawaiian fauna of fungus-feeding Phlaeothripinae is possibly diverse, but future studies need to be based on good population samples, rather than on a few isolated individuals.

One species is here excluded from the Hawaiian list. Hood (1939) placed *japonicus* Karny as a synonym of *flavipes* Bagnall, as is discussed below under the latter name. When this synonymy was subsequently rejected, the name *japonicus* seems to have been retained on the Hawaiian checklist, but without any recorded specimens. It is a member of the *fungi* species-complex as discussed below under *dubius*.

### Key to *Hoplothrips* of Hawaii

**Table:** 

1	All legs including coxae yellow	***H. flavipes***
–	Coxae and femora light brown to brown	**2**
2	Head with one or more stout cheek setae (Fig. 13); antennal segment VIII only weakly narrowed to base; pelta with posterolateral corners clearly curving away from anterior margin of tergite II (Fig. 16); antennal segment IV usually sharply bicoloured with basal third yellow and slender, at least 1.8 times as long as wide (Fig. 14)	**3**
–	Head with cheek setae weak and not prominent (Fig. 7); antennal segment VIII strongly narrowed to base, sometimes with distinct pedicel; pelta with posterolateral corners either confluent with anterior margin of tergite II (Figs 9, 10), or only very weakly curving away (Figs 18, 21); antennal segment IV either uniformly brown (Figs 12, 20), or if weakly bicoloured and increasingly pale toward almost yellow pedicel then scarcely 1.6 times as long as wide (Fig. 19)	**5**
3	Tergites III–IV with posteroangular setae at least 0.6 as long as median length of tergite; female with tergite IX setal pair S3 as long and slender as S1	***H. dubius***
–	Tergites III–IV with posteroangular setae less than 0.3 as long as median length of tergite (Fig. 23); female with tergite IX setal pair S3 no more than 0.6 as long as S1	**4**
4	Female with tergites VI–VIII lateral setae no more 0.3 as long as median length of these tergites (Fig. 24)	***H. lanaiensis***
–	Female with tergites VI–VIII lateral setae more than 0.6 as long as median length of these tergites	***H. perkinsi***
5	Female with pronotal anteroangular setae scarcely 0.5 as long as width of fore tibia; male sternite VIII apparently with no pore plate	***H. laticornis***
–	Female with pronotal anteroangular setae at least 1.5 times as long as width of fore tibia; male sternite VIII with transverse pore plate	**6**
6	Posterolateral corners of pelta rounded but close to anterior margin of tergite II (Fig. 18); pore plate on sternite VIII of male large, occupying about half of sternite area; female with antennal segment IV paler on basal third (Fig. 19); tergites II–VII of macropterae each with two pairs of curved or sigmoid wing-retaining setae	***H. flavitibia***
–	Posterolateral corners of pelta drawn out along anterior margin of tergite II (Figs 3, 9, 10); pore plate on sternite VIII of male slender and transverse; female with antennal segment IV brown (Fig. 12); tergites III–VII of macropterae each with only posterior pair of wing-retaining setae curved or sigmoid [large male with tubercles behind eyes (Fig. 1), and fore tibia with tubercle at inner apex]	***H. magnaccai* sp. n.**

#### Hoplothrips dubius

Taxon classificationAnimaliaThysanopteraPhlaeothripidae

(Bagnall)

Dolerothrips dubius Bagnall, 1910: 691
Dolerothrips barbatus Bagnall, 1910: 683. **Syn. n.**
Dolerothrips ovatus Bagnall, 1910: 686. **Syn. n.**
Dolerothrips angusticeps Bagnall, 1910: 688. **Syn. n.**
Dolerothrips bicolor Bagnall, 1910: 688. **Syn. n.**
Hoplothrips coprosmae Moulton, 1936: 186. **Syn. n.**

##### Remarks.

This species is a member of the *Hoplothrips fungi* complex. This comprises *corticis*, *fungi*, *japonicus*, *karnyi*, *orientalis* and *ulmi*, and Stannard (1968: 459) suggested that these various names may refer to a single species that is widespread across the Holarctic; Schliephake and Klimt (1979) even placed *fungi* as a synonym of *ulmi*. However, as indicated below, each of these six species can, at present, be distinguished from the others. In contrast, *dubius* from the Hawaiian Islands cannot be distinguished satisfactorily from some individuals identified as *karnyi* from North America, including from Vancouver, Canada. In the absence of more extensive studies on the *Hoplothrips* fauna of Western North America, the name *dubius* seems appropriate to use for the form on Hawaii. Currently, the six species in the *fungi* complex are distinguished from each other as follows. Specimens with antennal segment IV brown are placed in *orientalis*, the other five all having antennal segments IV–V yellow at the base. The males of *fungi* share with those of *japonicus* and *orientalis* a particularly large pore plate on sternite VIII, but *fungi* is distinguished by having long slender sense cones on antennal segment III, particularly the one on the inner apical margin. According to Okajima (2006), *japonicus* has antennal segment III particularly elongate, more than 2.6 times as long as wide, although the sense cones on that segment are as short as in *ulmi*; in *orientalis*, these sense cones are intermediate in length between *fungi* and *ulmi*. Finally, *ulmi* has short sense cones on antennal segment III, and the median length of the pore plate on sternite VIII of males is much shorter than in *fungi* (Mound et al. 1976; Okajima 2006). Specimens identified as the North American species, *karnyi*, have the short antennal sense cones of *ulmi*, but the pore plate on sternite VIII of males is variable and intermediate between the condition found in *fungi* and *ulmi* from Europe The species here identified as *dubius* was found quite commonly on Hawaii, in Volcano National Park, in June 2016, and also at two sites on Oahu – Makuleia trail and Manoa Cliffs trail.

Bagnall described *dubius* from five winged females and one “aptera”, taken variously on the three islands – Hawaii, Molokai and Lanai. However, only the macropterous female from “Molokai Mts” remains, and this has only one antenna (Fig. 14) and is slide mounted and designated lectotype (Mound 1968). The unique holotype of *barbatus*, a large micropterous male from Kona, Hawaii, was described as lacking the distal antennal segments, although segments III and IV are available and are brown with the basal third yellow. Sternites III–VII have paired reticulate areas similar to those illustrated on the male syntype of *flavipes* (Fig. 22), and the median length of the pore plate on sternite VIII is about 30 microns. The lectotype of *ovatus* is the single large male that was taken on Haleakala, Maui, but of the original six females mentioned by Bagnall only two remain. The three specimens were slide mounted in 1967, and they retain antennae and most setae, but there is no reason to consider this as a different species from *dubius*. Antennae were not available on either of the two original specimens of *angusticeps*, and the lectotype is the male from Kalae, Molokai. Although much smaller than the *barbatus* holotype, it falls within the size range expected in species of *Hoplothrips* and has reticulate areas laterally on the sternites, and the pore plate on sternite VIII is about 40 microns long medially. Bagnall described *bicolor* from a single female taken on Kaala, Oahu. This lacked antennae, and the specimen is now slide mounted and lacks all major setae except for a single lateral seta on the third tergite; contrary to the original description, the tube is no paler than in other specimens here identified as *dubius*. Moulton based *coprosmae* on four females and six males from Nauhi, Hawaii. These specimens are in good condition, and they have been compared with macropterous and micropterous specimens of both sexes collected on Hawaii and Oahu in June 2016, all of which have variably prominent cheek setae on the head. The antennal sense cones are not as elongate as in *fungi* from Europe, and in males the sternites have reticulate areas laterally and the pore plate on sternite VIII is about 35 microns long medially. If these recent specimens, also the *coprosmae* types, had been collected in North America they would have been identified as *karnyi*. However, as indicated above, it is not possible at present to establish further synonymies between the fauna of Hawaii and that of the mainland until suitable studies are carried out on the North American *Hoplothrips* fauna.

#### Hoplothrips flavipes

Taxon classificationAnimaliaThysanopteraPhlaeothripidae

(Bagnall)

Dolerothrips flavipes Bagnall, 1910: 685.

##### Remarks.

This species was based on “several specimens… in alcohol” from Maui but with no date of collection; also “numerous specimens” (presumably dry and carded) from Maui on Mt. Haleakala in 1896. In the BMNH, only 1 male and 2 female micropterae remain of this species; these were slide mounted by Bagnall presumably from the series in alcohol, but without data apart from Maui. Similar specimens were sent to Hood (1939: 587) who claimed that the yellow legs were the result of storage in alcohol, and placed *japonicus* and *major* as synonyms of *flavipes*. Certainly *flavipes* is a member of the northern hemisphere *fungi* species-complex to which *japonicus* and *major* (a synonym of *karnyi*) belong. These species share the character states of a rather slender antennal segment VIII, an extra pair of discal setae on the metanotum, and the head with prominent cheek setae. However, because the coxae of the available *flavipes* specimens are also clear yellow, it is possible that the leg colour may be natural and not due to storage in ethanol. The identity and relationships of these specimens thus remain equivocal. They share many character states with *H. flavafemora* Okajima from southern Japan, but the available specimens are too poorly preserved to be sure that these two represent a single species. Moreover, if it were true that the pale legs of the *flavipes* specimens is due to storage in alcohol, then these specimens could not be distinguished from *dubius*. The male paralectotype is large, and laterally on sternites III–VII are extensive paired areas of iridescent reticulation (Fig. 22), and sternite VIII has a broad pore plate with a median length of about 35 microns. The female paralectotype is in particularly poor condition, but the lectotype female mounted onto a slide with the male has the lateral setae on tergites III–IV short, scarcely half as long as the tergite median length.

#### Hoplothrips flavitibia

Taxon classificationAnimaliaThysanopteraPhlaeothripidae

Moulton

Hoplothrips flavitibia Moulton, 1928: 117.

##### Remarks.

Moulton described this species from 45 specimens taken in 1927 on Waipio Ridge, Oahu. He compared this briefly to *japonicus* as well as *lanaiensis*, *laticornis* and *ovatus*. However, *flavitibia* shares with *corticis* from the northern hemisphere the following character states: rather short antennal segment III but slender VIII; metanotum without sculpture medially but with an additional pair of discal setae (Fig. 17); pelta posterolateral angles almost confluent with tergite II anterior margin (Fig. 18). At present there is insufficient material to establish a formal synonymy, but no obvious character states have been observed to distinguish *flavitibia* from *corticis*, a species that is widespread in Europe, North America and Japan, and also known from New Zealand. Zimmerman (1948) indicated that *flavitibia* had been found on Kauai, Maui, and Hawaii in addition to Oahu, and nine females with four males (in BMNH) have been studied that were collected and identified by Sakimura as *flavitibia* from Olinda, Maui. During July 2016, several specimens were taken near Volcano, Hawaii, and also on the Makuleia Trail, Oahu.

#### Hoplothrips lanaiensis

Taxon classificationAnimaliaThysanopteraPhlaeothripidae

(Bagnall)

Dolerothrips lanaiensis Bagnall, 1910: 690.
Hoplothrips hawaiiensis Moulton, 1936: 185. **Syn. n.**

##### Remarks.

In describing *hawaiiensis* from eight females and two males (presumably all micropterae) taken on Oahu and Maui, Moulton compared it to *perkinsi*, claiming that this was the only species from Hawaii with “spines so reduced at the posterior angles of abdominal segments”. However, Bagnall clearly stated of *lanaiensis* “abdominal bristles obsolete” when he described this species from 10 female and 8 male micropterae taken on Lanai, Molokai and Hawaii. The tergal lateral setae on the only remaining specimens of this species (two females and the lectotype male from Lanai) are similar to those on *hawaiiensis* (Figs 23, 24). As in *perkinsi*, the metanotum is reticulate medially. However, in *perkinsi* the lateral setal pair on tergites VI–VIII are longer (on VII 180 microns) than on the type specimens of *lanaiensis* and *hawaiiensis* (on VII no more than 30 microns). The published year of collection differs for the type specimens of *perkinsi* and *lanaiensis*, but their collection numbers (Perkins 91 and 92) suggest that they were actually collected together at the same locality on Lanai. If more specimens become available, the differences in tergal setal lengths may be found to fall within the range of a single species, and *lanaiensis* would thus be a synonym of *perkinsi*. However, a single macropterous female was collected on Oahu, Mokuleia Trail, in July 2016 that is here identified as *lanaiensis*. The pronotal epimeral setae, and also setae S1 and S2 on tergite VIII, are short with curiously blunt apices.

#### Hoplothrips laticornis

Taxon classificationAnimaliaThysanopteraPhlaeothripidae

(Bagnall)

Trichothrips laticornis Bagnall, 1910: 692.
Hoplothrips mauiensis Moulton, 1928: 119. **Syn. n.**

##### Remarks.

Bagnall described this species from a single, slide-mounted, macropterous female collected at Kona, Hawaii, in 1892. This specimen is mounted ventral side uppermost, with the wings folded on the body. Moulton described *mauiensis* from 21 specimens taken at Olinda, Maui in 1926 and 1927. This species appears to be diagnosed by the following character states: antennal segment VIII slender and narrowed to base, segment III no more than 1.6 times as long as wide, segment IV not sharply paler in basal third and no more than 1.5 times as long as wide; pronotal anteroangular setae no more than 30 microns long; metanotal median area without sculpture, one pair of rather short median setae; pelta lateral lobes weakly curving away from anterior margin of tergite II; tergite IX with three pairs of setae about 0.8 as long as tube. The only available male of *mauiensis* appears to lack a pore plate on sternite VIII, but this is possibly an artefact due to poor slide preparation. The relatively short and broad, almost uniformly coloured, fourth antennal segment (Fig. 20) does not seem to have been reported in any other member of this genus from any part of the world, although an almost similar condition exists in a few specimens identified as *flavitibia*.

#### Hoplothrips magnaccai sp. n.

Taxon classificationAnimaliaThysanopteraPhlaeothripidae

http://zoobank.org/C4F3EDFE-638E-4DEC-9048-0839D3DCF4EC

##### Description.

**Figures 1–6.**
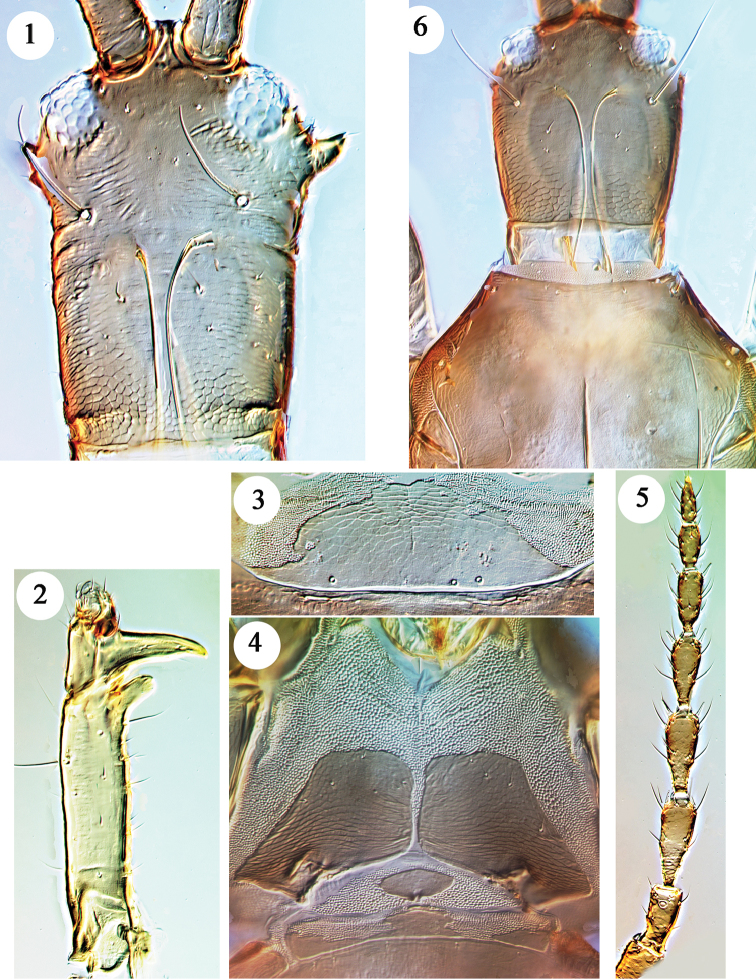
*Hoplothrips magnaccai*, males. Holotype **1–5**: **1** head **2** fore tibia and tarsus **3** pelta **4** prosternites **5** antenna **6** Small male head and pronotum.

***Male microptera.*** Head yellowish-brown and darkest around antennal bases; fore legs yellowish-brown, prothorax and pterothorax brown, abdomen paler, tube with yellow sub-apical area; mid and hind femora brown, tibiae and tarsi yellowish; antennal segment I brown, apex of II and basal half of III yellow, rest of antenna brown; major setae pale. Head slender, twice as long as width at base, with prominent lateral tubercles behind small eyes (Fig. 1); ocelli absent; postocular setae long and acute, longer than half of head width; maxillary stylets retracted to postocular setae, close together medially; ventrally all setae small, frons of largest male with pair of large irregular tubercles, absent in small male; mouth cone short and rounded. Antennae 8-segmented (Fig. 5); major male with segment I exceptionally long; VIII narrowed to small pedicel; 3 sense cones on III, 4 on IV, sense cone length little more than half of segment width. Pronotum massive, median longitudinal apodeme weak; anteromarginal setal pair very small, posteroangular pair unusually long in smaller male; prosternal basantra absent (Fig. 4), ferna large with median margins parallel in largest male but rounded in smaller males; mesopresternum of three weakly joined small sclerites; metathoracic sternopleural sutures present but short. Fore femora elongate, extending to apical margin of head in largest male, but not beyond mid-point of head in smaller males; fore tibia with large, broadly rounded tubercle at inner apex dorsal to the normal apical seta (Fig. 2); fore tarsal tooth as long as tarsal width. Meso and metanota transverse, metanotum without sculpture medially; fore wing lobe 50 microns long, bearing one seta. Pelta broadly D-shaped (Fig. 3), posterior margin confluent with anterior margin of tergite II; tergites II–VII each with 2 pairs of short, straight wing-retaining setae; tergite IX setae shorter than tube, tube much shorter than head. Sternite VIII with slender pore plate, median length about 15 microns, extending fully across sternite; median sternites without any lateral reticulate areas.

Measurements (holotype male and smallest paratype male in microns). Body length 3500 (2900). Head, length 380 (250); width posterior to tubercles 230 (215); po setae 125 (135). Pronotum, length 500 (280); width 500 (350); major setae: aa 20 (30), ml 100 (100), epim 120 (130), pa 180 (185). Tergite IX setae, S1 180 (185), S2 75 (70), S3 180 (160). Tube length 240 (215). Antennal segments I–VIII length 100 (65), 75 (60), 115 (85), 105 (85), 90 (70), 80 (?), 60 (?), 60 (?).

***Female microptera.*** Body and femora brown, basal half of head paler than pronotum, antennae brown except for base of segment III (Fig. 12), tibiae and tarsi shading yellowish-brown to yellow. Head with convex cheeks (Fig. 11), constricted behind small eyes with small tubercle ventro-laterally just behind eyes; ocelli absent; po setae long and acute; maxillary stylets close together medially and retracted to eyes. Pronotum transverse, ml, epim and pa setae long. Prosternal ferna large, narrowing medially; mesopresternum of three small sclerites. Tergites similar to those of male microptera.

**Figures 7–12.**
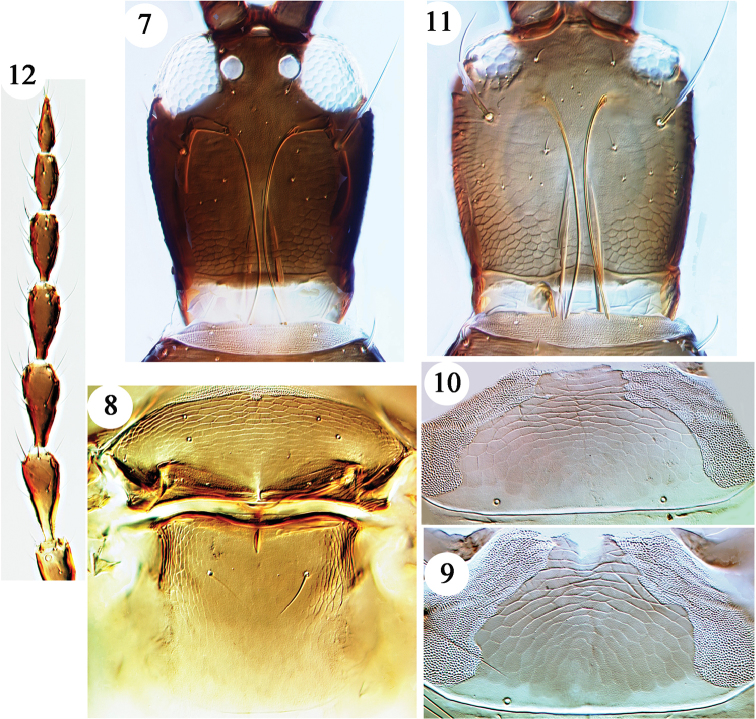
*Hoplothrips magnaccai*, females. Macroptera **7–9**: **7** head **8** meso and metanota **9** pelta. Microptera **10–12**: **10** pelta **11** head **12** antenna.

***Female macroptera.*** Darker than microptera, head dark brown (Fig. 7) and darker than pronotum, mid and hind tibiae mainly brown; fore wings pale. Head with cheeks convex, slightly constricted behind large eyes, without any tubercles; ocelli large, stylets retracted to eyes. Body similar to microptera; metanotum with no sculpture medially (Fig. 8); mesopresternum almost entire with three sclerites joined; fore wing with only two pairs of long sub-basal setae; tergites III–VII each with only one pair of sigmoid wing-retaining setae, anterior pair short and straight on these tergites, and both pairs short and straight on tergite II.

Measurements (macropterous female paratype in microns). Body length 3200. Head, length 300; width 250; po setae 140. Pronotum, length 230; median width 350; major setae: aa 15, am 75, ml 140, epim 130, pa 190. Fore wing length 1250; sub-basal setae 80. Posteroangular tergal setae: tergite II 30, tergite VI 180. Tergite VIII setae S1 180, S2 210, S3 240. Tube length 250. Antennal segments I–VIII length 60, 68, 100, 95, 90, 85, 60, 60.

##### Material studied.

Holotype male microptera, **OAHU**, Mokuleia Trail, from dead branches, 29.vii.2016 (LAM 6310), in BPBM, Hawaii.

Paratypes: 2 female macropterae, 6 female micropterae taken with holotype; at same site and date, 25 female macropterae (many de-alate), 3 female micropterae, 2 male micropterae (A. Wells 83, 84, 86, 87). **MAUI**, Io’a Needle, 2 female micropterae from dead branches, 26.vii.2016 (A.Wells 77).

##### Comments.

The macropterae of *magnaccai* are particularly unusual among *Hoplothrips* species, in that on tergite II both pairs of wing-retaining setae are small and straight and on each of tergites III–VII only the posterior pair is sigmoid with each anterior pair short and straight. Moreover, there are only two long sub-basal setae on each fore wing. In large males, the head of *magnaccai* is similar in appearance to that of two species known only from eastern USA: *Hoplothrips flavicauda* (Fig. 25) from several northeastern states (Stannard 1968), and *Hoplothrips mutabilis* from Florida (Hood 1955). In each of these three species, the largest short-winged males have a prominent tubercle behind the eyes, although such tubercles are not present in winged males (where known) nor in smaller short-winged males. The species most closely similar to *magnaccai* seems to be *mutabilis*, because these two share with typical *Hoplothrips* species both the absence of prosternal basantra (= praepectus of Stannard, 1968) and the presence in males of a transverse pore plate on sternite VIII. In contrast, both *flavicauda* and also *Hoplothrips fungosus* Moulton from eastern Asia (Okajima 2006) are distinctive within the genus *Hoplothrips* for the presence of prosternal basantra (Fig. 26), and the absence in males of a pore plate on sternite VIII. This new species differs from *mutabilis* as follows: body and first two antennal segments brown to dark brown rather than mainly yellow, postocular setae longer in all morphs, fore wing with about 15 duplicated cilia rather than eight, and sense cones of macropterae not long and slender.

**Figures 13–21.**
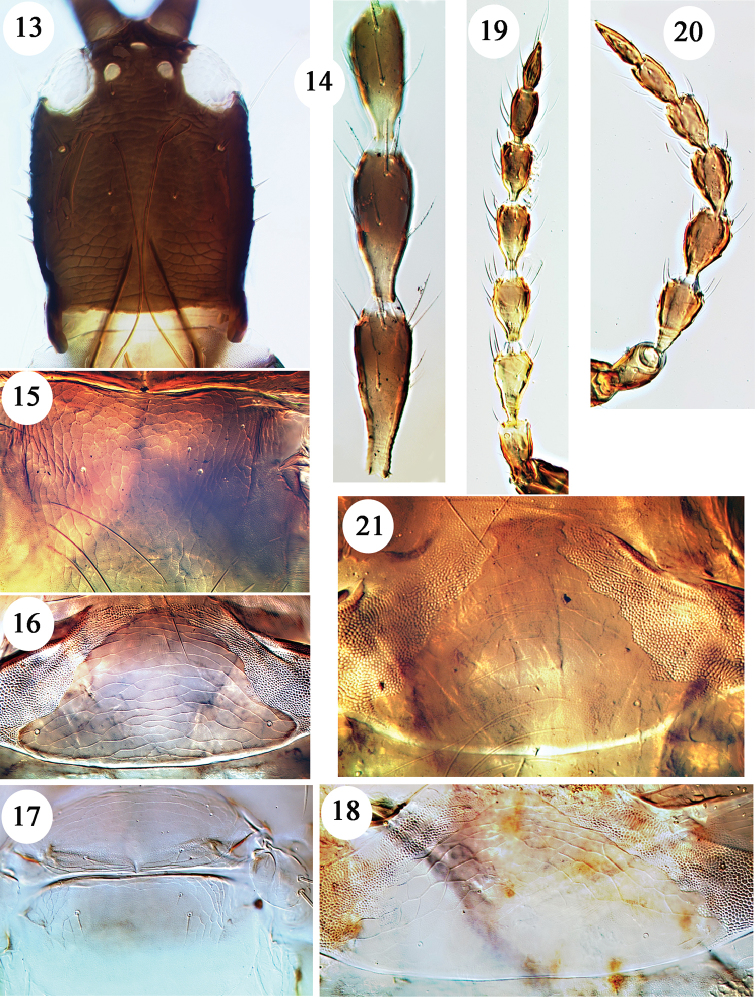
*Hoplothrips* species. *H. dubius* **13–16**: **13** female head **14** lectotype antennal segments III–V **15** lectotype metanotum **16** lectotype pelta. *H. flavitibia* paratype female **17–19**: **17** meso and metanota **18** pelta **19** antenna. *H. laticornis* holotype **20–21**: **20** antenna **21** pelta.

**Figures 22–26.**
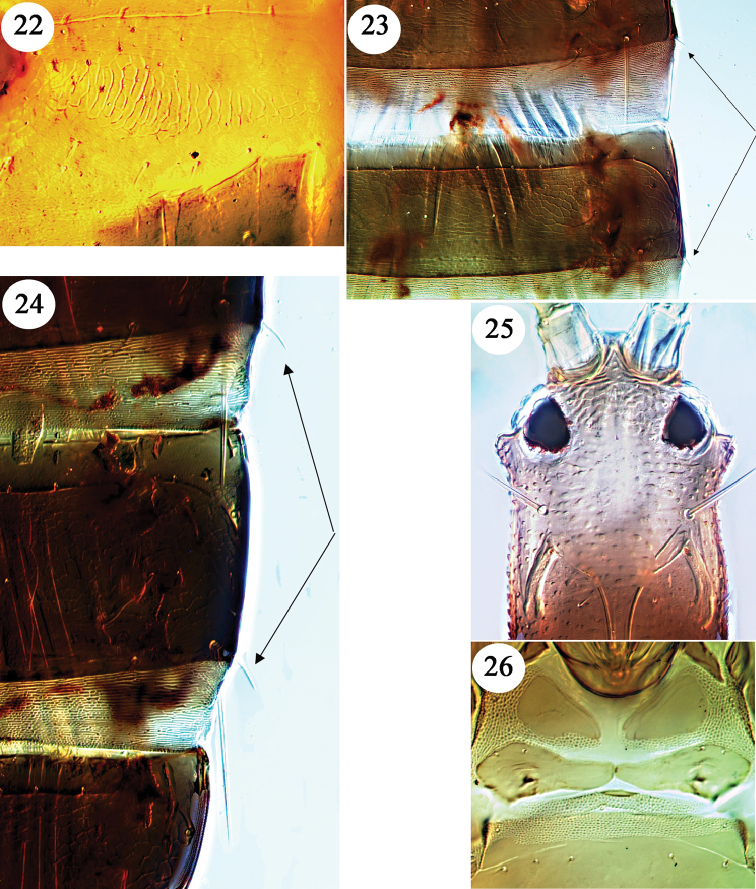
*Hoplothrips* species. **22** *H. flavipes* paralectotype male, sternite V. *H. hawaiiensis* paratype female **23–24**: **23** tergites II–III **24** tergites VI–VII. *H. flavicauda* 25–26: **25** large male microptera, head **26** female macroptera prosternites.

#### Hoplothrips perkinsi

Taxon classificationAnimaliaThysanopteraPhlaeothripidae

(Bagnall)

Dolerothrips perkinsi Bagnall, 1910: 687.
Dolerothrips intermedius Bagnall, 1910: 689. **Syn. n.**
Trichothrips nigricans Bagnall, 1910: 693. **Syn. n.**
Hoplothrips swezeyi Moulton, 1928: 120. **Syn. n.**

##### Remarks.

Described from a single female taken on Lanai, this specimen was described as having the antennae dark brown with only segment III yellow at base, but no antennae were found when the holotype was slide mounted in 1967. Another species described by Bagnall from Hawaii that has antennal segment IV almost brown is *laticornis*, but that has the metanotal median area without sculpture, whereas in *perkinsi* this area is distinctly reticulate. However, *intermedius* was described from a single major male taken on Haleakala, Maui, and although the slide mounted holotype lacks the tube, the single remaining antenna has segment IV brownish-yellow on the basal third. Unfortunately, *nigricans* was described from a single winged female that lacked antennae, and the slide mounted holotype now lacks most major setae apart from one long lateral seta on tergite VIII. In contrast, Moulton described *swezeyi* from 12 females and four males taken at Olinda, Maui, and distinguished this from *intermedius* by “its differently colored antennae”. Micropterous females of *swezeyi* share with *perkinsi* a distinctly reticulate metanotum, also long lateral setae on tergites VI–VIII but with short lateral setae on tergites III and IV, and the largely brown antennal segment IV with the basal third brownish-yellow. The statement by Bagnall that *perkinsi* has “obsolete” lateral setae on tergite VIII is not correct, and was presumably based on examining the holotype dry on a card. In females, tergite IX setae S3 are unusually short, less than 0.6 as long as setae S1, the dorsal pair. The holotype of *intermedius*, also males of *swezeyi*, have reticulate areas laterally on sternites III–VII, and the median length of the pore plate on sternite VIII is about 35 microns. Apart from the differences in setal lengths, *perkinsi* and *dubius* are similar in many details, and unlike the other species considered here, the heads of both sexes and both morphs of these species have quite prominent cheek setae.

## Supplementary Material

XML Treatment for Hoplothrips dubius

XML Treatment for Hoplothrips flavipes

XML Treatment for Hoplothrips flavitibia

XML Treatment for Hoplothrips lanaiensis

XML Treatment for Hoplothrips laticornis

XML Treatment for Hoplothrips magnaccai

XML Treatment for Hoplothrips perkinsi
